# CD4+ T Cells Targeting Dominant and Cryptic Epitopes from *Bacillus anthracis* Lethal Factor

**DOI:** 10.3389/fmicb.2015.01506

**Published:** 2016-01-05

**Authors:** Stephanie Ascough, Rebecca J. Ingram, Karen K. Y. Chu, Julie A. Musson, Stephen J. Moore, Theresa Gallagher, Les Baillie, Ethel D. Williamson, John H. Robinson, Bernard Maillere, Rosemary J. Boyton, Daniel M. Altmann

**Affiliations:** ^1^Avian Viral Immunology Group, The Pirbright InstitutePirbright, UK; ^2^Centre for Infection and Immunity, Queen’s University BelfastBelfast UK; ^3^Section of Infectious Diseases and Immunity, Department of Medicine, Imperial College LondonLondon, UK; ^4^Institute of Cellular Medicine, Newcastle UniversityNewcastle upon Tyne, UK; ^5^Center for Biomedical Engineering and Technology, University of Maryland School of MedicineBaltimore, MD, USA; ^6^School of Pharmacy and Pharmaceutical Sciences, Cardiff UniversityCardiff, UK; ^7^Defence Science and Technology LaboratorySalisbury, UK; ^8^Service d’Ingénierie Moléculaire des Protéines, Insititut de Biologie et de Technologies de Saclay, Commiseriat à l’Energie Atomique, Gif Sur YvetteFrance

**Keywords:** anthrax, lethal factor, epitope, cryptic, subdominant, immunodominant, HLA, CD4+

## Abstract

Anthrax is an endemic infection in many countries, particularly in the developing world. The causative agent, *Bacillus anthracis*, mediates disease through the secretion of binary exotoxins. Until recently, research into adaptive immunity targeting this bacterial pathogen has largely focused on the humoral response to these toxins. There is, however, growing recognition that cellular immune responses involving IFNγ producing CD4+ T cells also contribute significantly to a protective memory response. An established concept in adaptive immunity to infection is that during infection of host cells, new microbial epitopes may be revealed, leading to immune recognition of so called ‘cryptic’ or ‘subdominant’ epitopes. We analyzed the response to both cryptic and immunodominant T cell epitopes derived from the toxin component lethal factor and presented by a range of HLA-DR alleles. Using IFNγ-ELISpot assays we characterized epitopes that elicited a response following immunization with synthetic peptide and the whole protein and tested their capacities to bind purified HLA-DR molecules *in vitro*. We found that DR1 transgenics demonstrated T cell responses to a greater number of domain III cryptic epitopes than other HLA-DR transgenics, and that this pattern was repeated with the immunodominant epitopes, as a greater proportion of these epitopes induced a T cell response when presented within the context of the whole protein. Immunodominant epitopes LF_457-476_ and LF_467-487_ were found to induce a T cell response to the peptide, as well as to the whole native LF protein in DR1 and DR15, but not in DR4 transgenics. The analysis of Domain I revealed the presence of several unique cryptic epitopes all of which showed a strong to moderate relative binding affinity to HLA-DR4 molecules. However, none of the cryptic epitopes from either domain III or I displayed notably high binding affinities across all HLA-DR alleles assayed. These responses were influenced by the specific HLA alleles presenting the peptide, and imply that construction of future epitope string vaccines which are immunogenic across a wide range of HLA alleles could benefit from a combination of both cryptic and immunodominant anthrax epitopes.

## Introduction

The acute zoonotic disease, anthrax, caused by the Gram-positive bacterium *Bacillus anthracis*, is endemic or hyperendemic in a number of regions, particularly developing countries in sub-Saharan Africa, South America, and Asia (Dixon et al., 1999). The disease primarily affects grazing animals, with sporadic outbreaks in humans usually confined to agricultural workers and individuals in contact with the skins and fibers of infected animals (Wattiau et al., 2009). Anthrax has historically garnered attention because of its efficacy as a bioweapon, due to a high case fatality rate, limited person-to-person spread and the formation of spores that can be stockpiled in a weaponized form for prolonged periods. This was demonstrated in 2001, when anthrax spores were deliberately disseminated in the United States mail, resulting in a total of 22 cases of anthrax, with a 45% fatality rate in patients with inhalational disease, despite appropriate antibiotic therapy (Jernigan et al., 2002). More recently, anthrax outbreaks have been reported in persons who inject drugs (PWIDs) in Northern Europe during 2009–2010 and 2012–2013 (Ramsay et al., 2010; Ascough et al., 2014a; Berger et al., 2014; Ascough and Altmann, 2015). These cases involved the injection of heroin, contaminated with anthrax spores, directly into subcutaneous tissue. It is currently believed that a single *B. anthracis* strain type, closely related to strains from Turkey, was responsible for the outbreaks, and may have been introduced through contact with animal skins used to smuggle the heroin into Europe (Price et al., 2012; Grunow et al., 2013). These cases have illustrated that anthrax still remains an environmental hazard in many parts of the developing world, capable of impacting upon the health of people over a wide geographic range.

The main virulence factors of *B. anthracis* are encoded by genes found on two large plasmids. The structural genes which code for toxin production are carried on the 184.5 kbp plasmid, pXO1 and the formation of a poly-γ-D-glutamic acid capsule is directed by genes found on the 95.3 kbp plasmid, pXO2 (Duesbery and Vande Woude, 1999). Three of the genes found on pXO1; pagA, lef and cya, are responsible for encoding the three toxins associated with pathogenicity of *B. anthracis*; Protective Antigen (PA), Lethal Factor (LF) and Edema Factor (EF), respectively. The PA protein binds to either EF or LF to form the binary exotoxins Edema Toxin (ET), or Lethal Toxin (LT; Liu et al., 2014). Through binding to cell surface receptors, these toxins are endocytosed into the cytoplasm, where they affect the survival and multiplication of the bacilli within the host, subverting host immunity (Duesbery and Vande Woude, 1999).

The current licensed human vaccines, anthrax vaccine precipitated (AVP) and anthrax vaccine adsorbed (AVA or Biothrax), are both comprised of filtered supernatants from cell cultures of pXO1+/pXO2– *B. anthracis* strains, containing variable concentrations of the anthrax toxins (Williamson and Dyson, 2015). Ongoing efforts to develop more rigorously defined ‘next generation’ vaccines demand the characterisation of immune responses to potential antigens (Altmann, 2015). Despite the preponderance of studies that focus on the importance of PA in developing vaccination strategies against anthrax infection, it has lately become clear that LF may represent a major target not only for antibody responses, but also T cell immunity in naturally exposed individuals. Our previous work has shown that epitopes identified from domains II and IV of LF dominate a Th1 associated IFNγ response, and are presented to CD4+ cells in the context of a broad range of HLA DR molecules (Ingram et al., 2010; Ascough et al., 2014b).

Antigens, such as the mature secreted LF protein, which is composed of 776 amino acid residues, typically possess a large number of possible immunogenic peptide sequences, but the cellular immune response focuses on a relatively restricted number of determinants, which contribute to the development of a T cell response against epitopes processed from the whole protein antigen. Epitopes capable of triggering this kind of potent T cell response, following *in vivo* priming with either the whole protein or peptide antigens, are classified as immunodominant. In contrast, epitopes that are normally hidden from T-cell recognition, but are immunogenic in peptide form, are termed cryptic or subdominant epitopes. Initial studies of this phenomenon concentrated on model antigens such as hen egg lysozyme (Maizels et al., 1980; Adorini et al., 1988a,b; Nelson et al., 1992; Sercarz et al., 1993; Viner et al., 1995), the most intensive study of cryptic epitopes in disease has been carried out within the context of determinant spreading in autoimmunity, during which autoreactive T cell epitope recognition expands from a limited number of immunodominant epitopes to numerous cryptic epitopes (Lehmann et al., 1992; Sercarz, 1998; Vanderlugt and Miller, 2002; McMahon et al., 2005; Ji et al., 2013). However, recent work has suggested that the incorporation of subdominant or cryptic epitopes into vaccines, alongside immunodominant T cell epitopes, may have a major advantage in aiding the development of an immune response against microbial pathogens (Dominguez et al., 2011). The T cell repertoire of the immune response following immunization and infection has proven to differ in regard to immunodominant epitopes, and our own studies in humans have demonstrated that exposure to live *B. anthracis* bacteria uncovers epitopes within LF, which were not identified as T cell determinants following protein antigen vaccination (Ingram et al., 2010). This suggests that crypticity status of epitopes varies between exposure to living pathogens and protein vaccination, so that such responses can come to play an essential role in directing the immune response to pathogens *in vivo*. Dominguez et al. (2011) found that immunization with cryptic and subdominant determinants from the protozoan parasite *Trypanosoma cruzi* contribute to an artificially broadened CD8+ repertoire that provides increased protection against infection, compared to immunization with the single immunodominant epitope. Woodworth et al. (2014) demonstrated the ability of cryptic epitope specific T cells to resist lymphocyte terminal differentiation during *Mycobacterium tuberculosis* infection, maintaining polyfunctionality and enhanced antigen specific proliferation. There is evidence that CD4+ T cells directed against cryptic epitopes provide enhanced protection against pathogen challenge (Aagaard et al., 2009), it is therefore conceivable that polyepitopic vaccines, containing both cryptic or subdominant and immunodominant epitopes, may act to broaden the peptide specificity of T cells, reducing the ability of pathogens to escape recognition by the immune system. Analysis of the antigen specific T cell response to LF, and identification of a range of immunogenic epitopes for incorporation into an epitope string vaccine may represent a more successful vaccination strategy.

## Materials and Methods

### HLA Transgenic Mice

HLA class II transgenic mice carrying genomic constructs for HLA products common in the human population; HLA-DRA1^∗^0101/HLA-DRB1^∗^0101 (HLA-DR1), HLA-DRA1^∗^0101/HLA-DRB1^∗^0401 (HLA-DR4), and HLA-DRA1^∗^0101/HLA-DRB1^∗^1501 (HLA-DR15) were crossed for more than six generations to C57BL/6 H2-Aβ^00^ mice, leading to lines lacking endogenous MHC class II molecules, these mice were generated and maintained as described previously (Ascough et al., 2014b).

Research was conducted in compliance with the Animals (Scientific Procedures) Act 1986 and UK legislation relating to experiments involving animals. All experiments were approved by local ethical review and the facility where this research was conducted was licensed by the Home Office.

### LF Antigens

A biologically inactive form of recombinant full-length LF (rLF) was produced in an *Escherichia coli* expression system as previously described (Baillie et al., 2004). A synthetic peptide panel, comprising of 20mer amino acids overlapping by 10 amino acids encompassing the full-length sequence of LF was purchased from Abgent, San Diego, CA, USA.

### LF Epitope Mapping

HLA transgenic mice were immunized subcutaneously with either individual immunodominant epitopes, or pools of non-overlapping LF peptides comprising domains III or I, (each pool containing ≤6 peptides) emulsified in Titermax Gold adjuvant (Sigma–Aldrich, USA). After 10 days, local draining popliteal lymph nodes were removed and disaggregated into single cell suspensions. Lymph node cells (3.5 × 10^6^/ml) were challenged with 25 μg/ml of either recombinant full-length LF, or individual LF peptides.

### IFNγ ELISpot Assays

Antigen-specific IFNγ levels from *ex vivo* murine T cell populations were analyzed by ELISpot (Diaclone), as previously described (Ascough et al., 2014b). In brief, ethanol pre-wetted hydrophobic PVDF membrane-bottomed 96-well plates (MAIP S 45; Millipore) were washed twice with PBS, then coated with anti-IFNγ monoclonal antibody at 4°C overnight. Plates were blocked with 2% skimmed milk, washed twice with PBS, and 100 μl/well of antigen was added in triplicate. Each assay included a medium only negative and a positive control of SEB (25 ng/ml). Wells were seeded with 100 μl of 3.5 × 10^6^cells/ml in HL-1 medium (1% L-Glutamine, 1% Penicillin Streptomycin, 2.5% β-Mercaptoethanol). Following incubation for 72 h at 37°C with 5% CO_2_, plates were washed twice with PBS Tween 20 (0.1%). Plates were incubated with biotinylated anti-IFNγ monoclonal antibody, then washed with PBS Tween 20 (0.1%). Incubation with streptavidin-alkaline phosphatase conjugate was followed by PBS Tween 20 (0.1%) washes and then treated with 5-bromo-4-chloro-3-indolyl phosphate and nitroblue tetrazolium (BCIP/NBT). Spot formation was monitored visually and plate contents were discarded. After washes with water, the plates were air-dried and incubated overnight at 4°C to enhance spot clarity. Spots were counted using an automated ELISpot reader (AID), and expressed as delta spot forming cells per 10^6^ cells (ΔSFC/10^6^; SFC/10^6^ of stimulated cells minus SFC/10^6^ of unstimulated control cells). The results were considered positive if the ΔSFC/10^6^ was more than two standard deviations above the mean of the control. Peptides were classed as positive epitopes if two or more out of three, three or more out of four, or four or more out of five immunized mice showed responses above this value.

### HLA Peptide Binding Assay

The relative binding affinity of LF peptides to HLA-DR molecules were determined by competitive ELISA, as previously described (Texier et al., 2000). Briefly, affinity chromatography was used to immunopurify HLA-DR molecules from homozygous EBV-transformed lymphoblastoid B cell lines. The HLA-DR molecules were diluted in binding buffer and incubated for 24–72 h with an appropriate biotinylated reporter peptide, and a serial dilution of the competitor LF peptides. The validity of each experiment was assessed by a control of unlabeled reporter peptides. Binding neutralization buffer was applied to the HLA-DR molecules and resultant supernatants were incubated at room temperature in ELISA plates (Nunc, Denmark) coated with monoclonal antibody L243. Bound biotinylated peptide was detected by addition of streptavidin-alkaline phosphatase conjugate (GE Healthcare, Saclay, France) and 4-methylumbelliferyl phosphate substrate (Sigma–Aldrich, France). A Gemini Spectramax Fluorimeter (Molecular Devices, St. Gregoire, France) was used to measure fluorescence at 450 nm post-excitation at 365 nM. Sequences of the reference peptide and their IC50 values were as follows: HA 306–318 (PKYVKQNTLKLAT) for DRB1^∗^0101 (4 nM), DRB1^∗^0401 (8 nM), and DRB1^∗^1101 (7 nM), YKL (AAYAAAKAAALAA) for DRB1^∗^0701 (3 nM), A3 152–166 (EAEQLRAYLDGTGVE) for DRB1^∗^1501 (48 nM), MT 2–16 (AKTIAYDEEARRGLE) for DRB1^∗^0301 (100 nM) and B1 21–36 (TERVRLVTRHIYNREE) for DRB1^∗^1301 (37 nM). LF peptide concentration that prevented binding of 50% of the labeled peptide (IC_50_) was determined, and data was expressed as relative binding affinity (ratio of IC_50_ of the LF competitor peptide to the IC_50_ of the reference peptide which binds strongly to the HLA-DR molecule). Strong binding affinity was defined as a relative activity <10.

## Results

### Immunization of HLA-DR Transgenic Mice with Domain III Peptides Unveils Cryptic Epitopes with an HLA Allele Specific Focus

Cryptic epitopes have relevance to infectious disease as responses to such epitopes may be unveiled as a consequence of antigen processing differences during infection. Our own studies in humans have demonstrated that environmental exposure to live *B. anthracis* uncovers cryptic epitopes within LF, not identified as T cell determinants following protein antigen vaccination (Ingram et al., 2010). This raises the possibility that cryptic epitopes may have a role in host-pathogen interplay in the pathogenesis of anthrax *in vivo*.

Immunodominant T cell epitopes from LF are largely localized to domains I, II and IV of LF. However, HLA binding affinity studies suggested additional potential epitopes within domain III not revealed by mapping the immunodominant epitopes. Domain III was of specific interest due both to its immunologically ‘silent’ nature, common to all HLA alleles during immunodominant epitope mapping, and the role that this region may play in dictating the stability and function of the LF catalytic site. We therefore attempted to identify cryptic CD4+ T cell responses to the peptides which encompass LF domain III in mice transgenic for three common HLA-DR alleles by immunizing with pools of non-overlapping LF peptides and restimulating lymph node cells with the individual domain III peptides.

**FIGURE 1:**
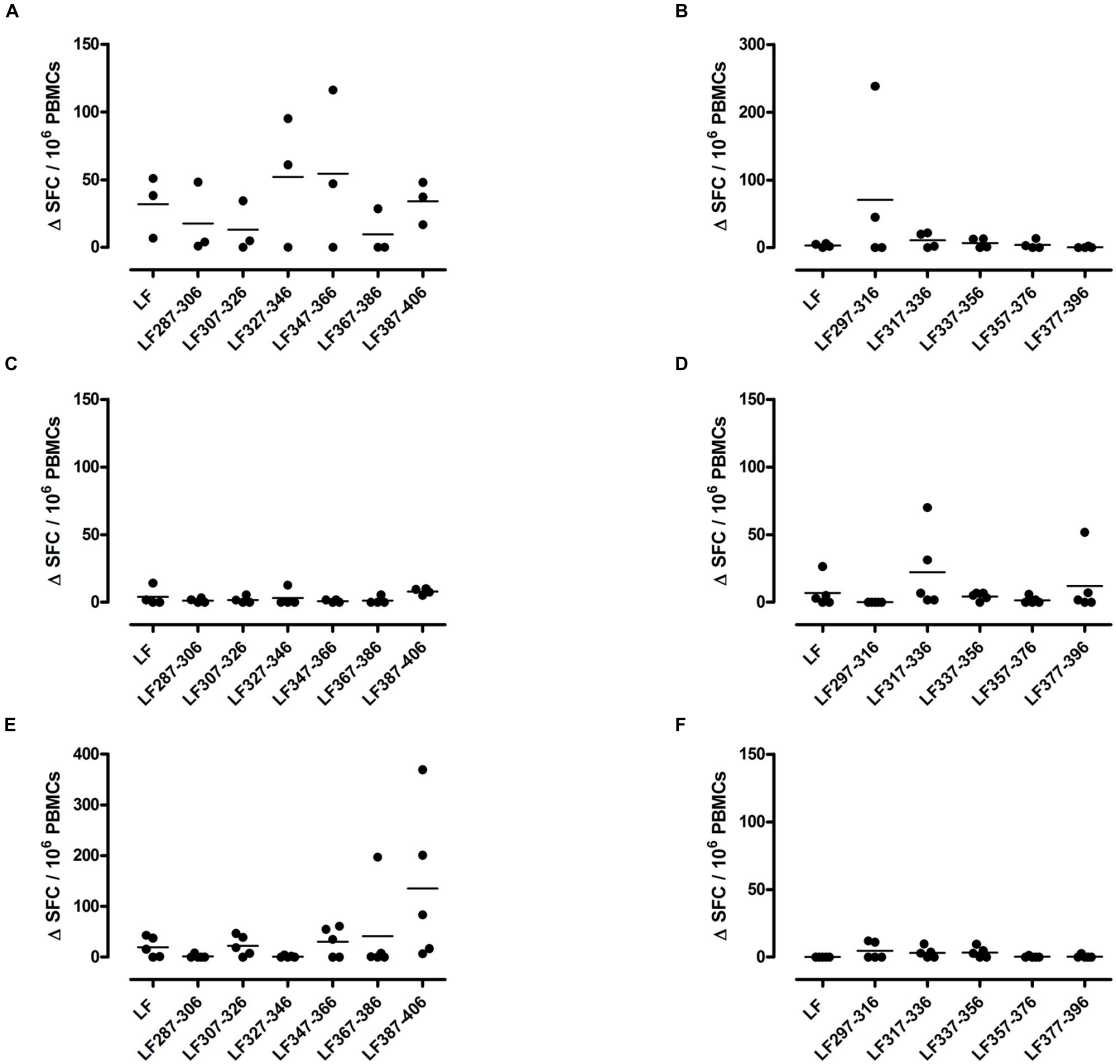
**HLA transgenic mice immunized with LF peptide pools comprising domain III generate HLA-DR restricted CD4+ T cell cryptic epitopes**. Transgenic mice; DR1 **(A,B)**, DR15 **(C,D)**, and DR4 **(E,F)** were immunized with pools designed to only include non-overlapping LF peptides comprising domain III. Draining lymph node cells were cultured in an IFNγ specific ELISpot plate with 25 μg/ml of the individual domain III LF peptides for 72 h. Results are given as the number of spots above background (ΔSFC)/10^6^ PBMCs with mean ± SD (*n* = 3–5 mice for each column).

A T cell recall response to the native protein as well as to the individual peptides was only induced in mice immunized with one peptide pool, HLA-DR1 transgenics demonstrated a T cell recall response to peptides LF_287-306_, LF_307-326_, LF_327-346_, LF_347-366_, and LF_387-406_ (**Figure 1A**), Only one HLA-DR15 restricted cryptic epitope was identified; LF_317-336_ (**Figure 1D**), while the HLA-DR4 restricted cryptic epitopes were identified as LF_307-326_ and LF_387-406_ (**Figure 1E**). Although, in comparison to immunodominant epitopes, lower levels of IFNγ were released from cells following stimulation with cryptic epitopes, all positive epitopes were found to produce a ΔSFC/10^6^ more than two standard deviations above the negative control mean value.

The largest number of LF domain III T cell epitopes were thus detected in the context of HLA-DR1 presentation. The cryptic epitopes LF_307-326_ and LF_387-406_ were common to both HLA-DR1 and HLA-DR4 transgenics, although only presentation by HLA-DR1 led to a recall response to the whole protein.

### Single Peptide Immunization of Immunodominant Epitopes Reveals Peptides Capable of Inducing a Response to the Native Protein

**FIGURE 2:**
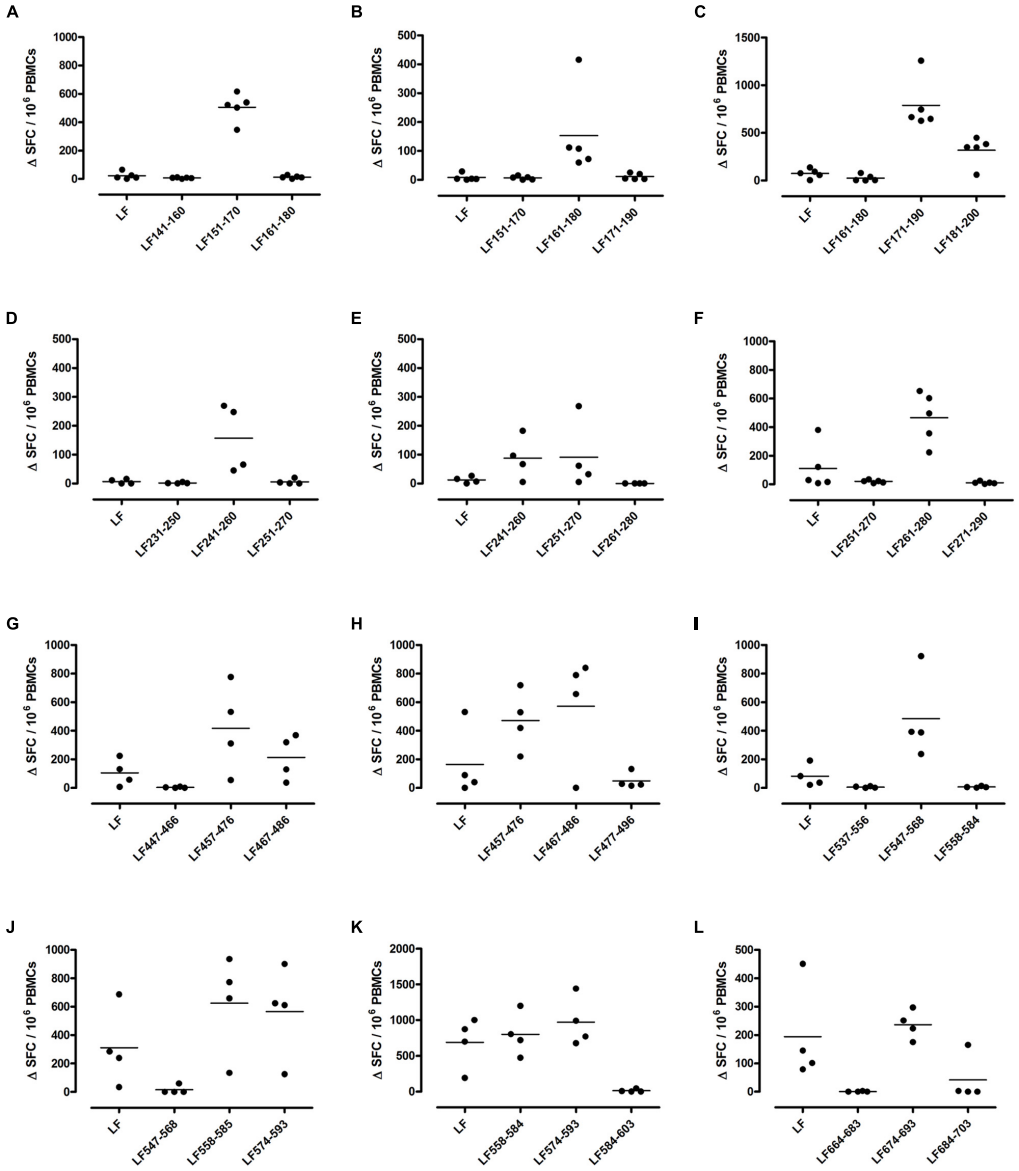
**Immunodominant HLA-DR1 restricted T cell epitopes**. HLA-DR1 transgenics were immunized with each of the immunodominant peptides separately **(A–L)**, and draining lymph node cells were cultured in an IFNγ specific ELISpot plate with the immunizing and flanking peptides or LF protein for 72 h. Results are given as the number of spots above background (ΔSFC)/10^6^ PBMCs with mean ± SD (*n* = 4–5 mice for each column).

**FIGURE 3:**
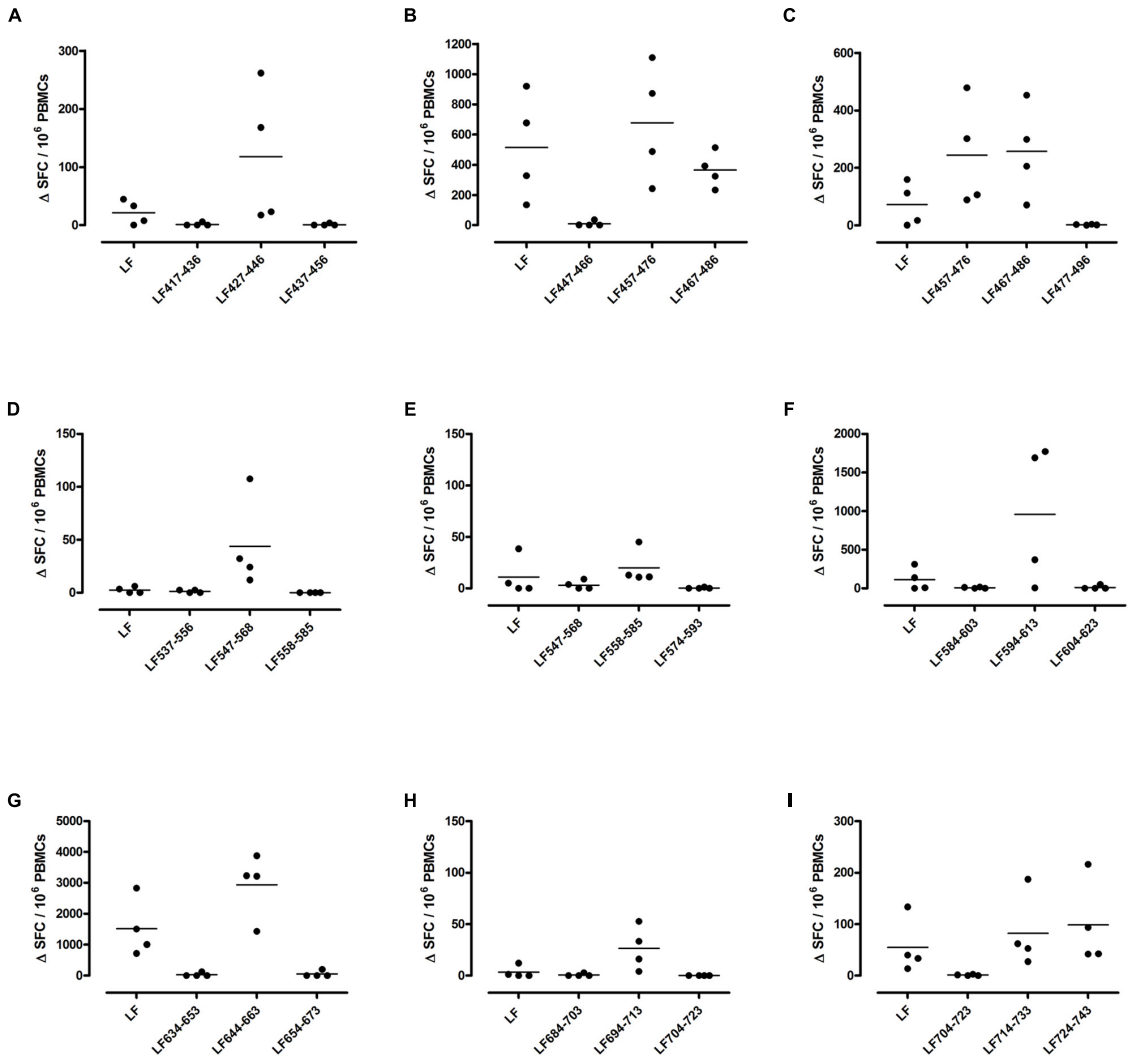
**Immunodominant HLA-DR15 restricted T cell epitopes**. HLA-DR15 transgenics were immunized with each of the immunodominant peptides separately **(A–I)**, and draining lymph node cells were cultured in an IFNγ specific ELISpot plate with the immunizing and flanking peptides or LF protein for 72 h. Results are given as the number of spots above background (ΔSFC)/10^6^ PBMCs with mean ± SD (*n* = 4–5 mice for each column).

**FIGURE 4:**
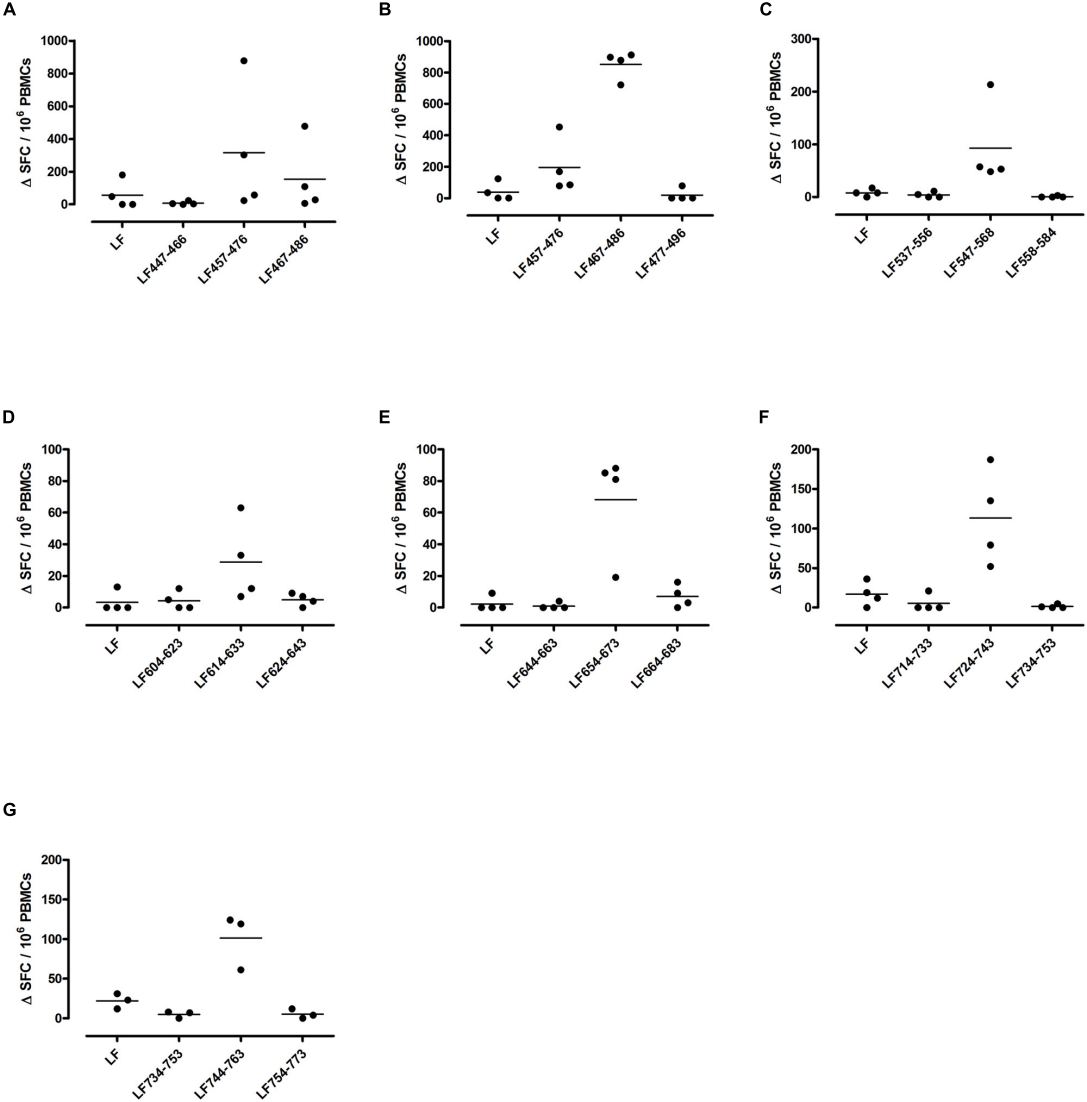
**Immunodominant HLA-DR4 restricted T cell epitopes**. HLA-DR4 transgenics were immunized with each of the immunodominant peptides separately **(A–G)**, and draining lymph node cells were cultured in an IFNγ specific ELISpot plate with the immunizing and flanking peptides or LF protein for 72 h. Results are given as the number of spots above background (ΔSFC)/10^6^ PBMCs with mean ± SD (*n* = 3–4 mice for each column).

Different MHC class II alleles present largely different peptide epitopes from a given antigen, shaping the T cell repertoire activated in the immune response. This diversity was observed here in the range of LF peptides presented by the HLA-DR transgenics. In addition to allele specific epitopes, our previous work reported that the domain II peptides LF_457-476_ and LF_467-487_, as well as the domain VI peptide LF_547-568,_ were all promiscuous immunodominant epitopes, capable of eliciting a T cell response from all the HLA-DR transgenic mice studied (Ascough et al., 2014b). The heteroclitic nature of these peptides appears to be dependent upon the presenting HLA-DR allele, as LF_457-476_ and LF_467-487_ were capable of inducing a T cell response to the peptide, as well as to the whole native LF protein in DR1 (**Figures 2G,H**) and DR15 (**Figures 3B,C**), but not in DR4 (**Figures 4A,B**) transgenics. In contrast, the response to immunization with LF_547-568_ did not lead to a response to the peptide within the context of the whole protein, regardless of which HLA-DR allele was presenting it.

DR1 transgenics demonstrated T cell responses to a greater number of domain III cryptic epitopes than other HLA-DR transgenics, and we found that this pattern was repeated with the immunodominant epitopes. Relative to other HLA-DR transgenics the DR1 repertoire was found not only to consist of a greater number of immunodominant epitopes, but also, a greater proportion of these epitopes; LF_171-190_, LF_457-476_, LF_467-487_, LF_558-584_, LF_574-593_, and LF_674-693_, induced a T cell response when presented within the context of the whole protein (**Figure 2**), compared to only four LF epitopes; LF_457-476_, LF_467-487_, LF_644-663_, and LF_714-733_, in DR15 (**Figure 3**) and none in DR4 transgenics (**Figure 4**).

### Cryptic Domain I Peptides are Capable of Inducing a Heteroclitic Response in HLA-DR4 Transgenics

**FIGURE 5:**
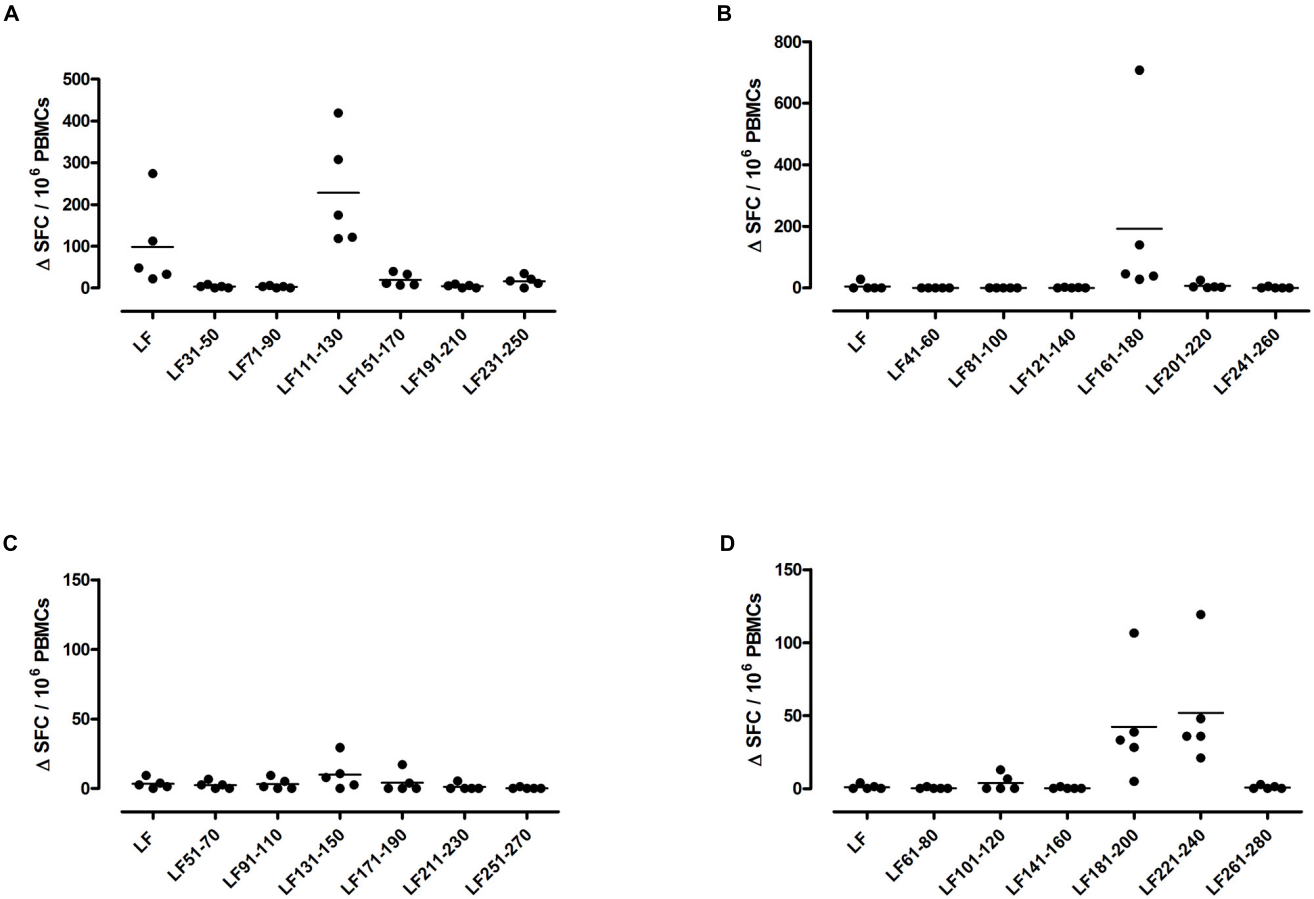
**HLA-DR4 transgenics immunized with LF peptide pools comprising domain I of the protein demonstrate CD4+ T cell cryptic epitopes**. HLA-DR4 transgenic mice were immunized with pools of non-overlapping LF peptides comprising domain I **(A–D)**, and draining lymph node cells were cultured in an IFNγ specific ELISpot plate with 25 μg/ml of each individual peptide from the immunizing pool. Results are given as the number of spots above background (ΔSFC)/10^6^ PBMCs with mean ± SD (*n* = 5 mice for each column).

A feature of both the domain III cryptic epitopes (**Figure 1**) and the immunodominant epitopes spread across domains II and IV (**Figure 4**) in HLA-DR4 transgenics, was that they were unable to induce a T cell response to the whole native LF protein. To determine whether this was an inherent characteristic of the HLA-DR4 restricted presentation of LF epitopes, we further screened peptides from domain I, which does not contain any immunodominant HLA-DR4 restricted epitopes, for the presence of heteroclitic cryptic epitopes. This revealed the presence of several unique epitopes, which had not been identified during the mapping of immunodominant epitopes within this domain. LF_111-130_, LF_161-180_, LF_181-200_, and LF_221-240_ all elicit T cell responses following administration in peptide form (**Figure 5**). Furthermore, the peptide pool which contained LF_111-130_ was also found to be capable of inducing a response which encompassed the whole native LF protein.

These results suggest that the cryptic and immunodominant epitopes within the LF protein are influenced to a great extent by the allele of the HLA class II molecules responsible for peptide presentation.

### HLA Class II Binding of Cryptic Epitopes Across Distinct HLA-DR Polymorphisms

The overlapping 20-mers representing domains III (**Table 1**) and I (**Table 2**) of LF were evaluated for binding to seven common HLA-DR alleles, DRB1^∗^0101 (DR1), DRB1^∗^0401 (DR4), DRB1^∗^1101 (DR11), DRB1^∗^0701 (DR7), DRB1^∗^1501 (DR15), DRB1^∗^0301 (DR3) and DRB1^∗^1301 (DR13).

**Table 1:** HLA restricted cryptic epitopes identified within domain III with relative binding affinity.

LF peptide sequence	HLA-restriction	Relative binding affinity of peptide to HLA-DR molecules						
		DR1	DR3	DR4	DR7	DR11	DR13	DR15
^287^ LSLEELKDQRMLSRYEKWEK ^306^	DR1	31	**3**	>1250	1,414	17	**3**	18
^307^ IKQHYQHWSDSLSEEGRGLL ^326^	DR1, DR4	73	900	**2**	53	84	857	67
^317^ SLSEEGRGLLKKLQIPIEPK ^336^	DR15	149	>667	>1788	488	23	100	268
^327^ KKLQIPIEPKKDDIIHSLSQ ^346^	DR1	**0.2**	**2**	750	1,195	1,200	857	14
^347^ EEKELLKRIQIDSSDFLSTE ^366^	DR1	267	**0.4**	**9**	120	**9**	926	**0.2**
^387^ KELLNRIQVDSSNPLSEKEK^406^	DR1, DR4	146	**1**	**0**	36	274	1,714	**1**

*The relative binding affinity of peptides to HLA-DR molecules were expressed as a relative activity (ratio of the IC_50_ of the peptide to the IC_50_ of a reference peptide which binds strongly to the individual HLA II molecule). Peptides with a high relative binding affinity of <10 are indicated in bold, while moderate binding is characterized by a range of relative affinity varying from 10 to 100. Means were calculated from at least three independent measurements*.

**Table 2:** HLA-DR4 restricted cryptic epitopes identified within domain I with relative binding affinity.

LF peptide sequence	Relative binding affinity of peptide to HLA-DR molecules						
	DR1	DR3	DR4	DR7	DR11	DR13	DR15
^111^ GGKIYIVDGDITKHISLEAL ^130^	122	>1000	61	15	75	21	**6**
^161^ VLVIQSSEDYVENTEKALNV ^180^	1702	82	29	66	168	>172	958
^181^ YYEIGKILSRDILSKINQPY ^200^	69	**3**	19	**4**	**0.4**	**5**	43
^221^ LLFTNQLKEHPTDFSVEFLE ^240^	909	>76	**6**	51	148	>172	193

*The relative binding affinity of peptides to HLA-DR molecules were expressed as a relative activity (ratio of the IC_50_ of the peptide to the IC_50_ of a reference peptide which binds strongly to the individual HLA II molecule). Peptides with a high relative binding affinity of <10 are indicated in bold, while moderate binding is characterized by a range of relative affinity varying from 10 to 100. Means were calculated from at least three independent measurements*.

Within domain III (**Table 1**) the DR1 restricted epitopes LF_287-306_ and LF_327-346_ showed moderate and strong binding to HLA-DR1, respectively. LF_307-326_ and LF_387-406_, which were identified in the DR4 and DR1 transgenics, both showed strong binding to HLA-DR4, but only LF_307-326_ showed moderate binding to HLA-DR1. All of the DR4-restricted domain I cryptic epitopes (**Table 2**) identified in the transgenics showed a strong to moderate relative binding affinity to HLA-DR4 molecules of <100, with LF_221-240_ demonstrating the highest binding affinity. Whilst the LF_181-200_ peptide exhibited promiscuous binding of <100 to all the HLA-DR molecules assayed. However, none of the cryptic epitopes from either domain III or I displayed notably high binding affinities across all HLA-DR alleles assayed

## Discussion

Microbial antigen presentation through HLA class II is a crucial step in initiating adaptive immunity to infection, the considerable allelic diversity of these class II molecules influencing susceptibility to infection. HLA class II transgenic mice were here used to characterize HLA restricted T cell responses to immunogenic epitopes of the LF toxin secreted by *B. anthracis*. The value of such studies is that they offer reductionist tools for mapping the microbial epitopes likely to be presented to T cells in humans carrying the same HLA alleles.

The major variables that influence the immunodominance and crypticity of epitopes derived from an antigen such as LF, remain a focus of speculation. It has been suggested that intrinsic features within the protein molecule reduce the availability of particular epitopes, and efficient endosomal antigen processing may be hampered by flanking amino acids which either promote the enzymatic destruction of the determinant or hinder processing through slow or incomplete unfolding of the protein molecule (Moudgil et al., 1998; Musson et al., 2003, 2006). This is supported by studies indicating that immunodominant epitopes tend to cluster within regions which are either within or adjacent to protease-sensitive loops (Carmicle et al., 2007). The non-classical MHC class II heterodimer DM has also been implicated in directing the cryptic and immunodominant fate of epitopes, although it remains unclear whether this is due to the DM induced editing of the epitope repertoire to favor peptides which display a higher binding affinity for MHC molecules (Kropshofer et al., 1996; Nanda and Sant, 2000; Nanda and Bikoff, 2005). The critical role peptide binding plays in determining dominance hierarchies has also been demonstrated by studies showing a direct correlation between immunodominance and the binding affinities of the MHC/peptide complex (Lazarski et al., 2005; Weaver et al., 2008). We discovered that although a number of epitopes such as the HLA-DR4 restricted LF_307-326_ and LF_387-406_, showed a strong binding affinity to the relevant MHC class II molecule, the strength of HLA binding did not adequately predict the immunodominance or crypticity of LF peptides. The underlying mechanisms governing the epitope hierarchy as yet remain unclear, however, our results indicate that identifying cryptic as well as immunodominant epitopes located within the anthrax toxins may be relevant in the attempt to elicit protective immune responses toward *B. anthracis*.

Immunization with cryptic or subdominant determinants has been found to contribute to an broadened T cell repertoire which provides increased protection against infection, compared to immunization with a single immunodominant epitope (Dominguez et al., 2011). By exploring the subdominant or cryptic responses to domain III and domain I within the context of HLA transgenics, we were able to identify cryptic epitopes which could be incorporated into polyepitopic vaccines alongside immunodominant T cell epitopes. This is particularly pertinant to anthrax infection, as previous studies have established that the T cell repertoire following immunization and live anthrax infection differs in regard to the epitopes uncovered (Ingram et al., 2010). The possibility that infection works to skew the epitopes which are preferentially processed or presented, uncovering infection specific epitopes, raises the possibility that epitopes which are not immunodominant, but rather cryptic or subdominant, may be relevant to the design of epitope string vaccines.

Furthermore, immunization with peptides which elicit a T cell response to the peptide in the context of the whole protein has been correlated with survival following pathogen infection (Zhang et al., 2009); within our study cryptic and immunodominant epitopes capable of eliciting a strong T cell response following administration in peptide form alone, were identified. The epitope pool which contained the DR1-restricted cryptic domain III epitopes LF_287-306_, LF_307-326_, LF_327-346_, LF_347-366_, and LF_387-406_ and the pool containing the DR4-restricted cryptic domain I epitope LF_111-130_, were the only cryptic epitopes identified capable of inducing a response to the whole protein. In contrast, a number of immunodominant epitopes, both HLA-DR1 and DR15 restricted, were identified which were capable of inducing a response to the whole LF protein. This included LF_457-476_ and LF_467-487_, which, unlike the majority of epitopes which are unique to a particular HLA allele, were a feature of both DR1, DR15, and DR4 transgenic responses. Although it should be noted that these promiscuous epitopes were unable to provoke a response to the peptides within the context of the whole protein following presentation by HLA-DR4. In fact, only cryptic epitopes from domains I and III were found to lead to a heteroclitic response against the whole protein in DR4 transgenics, this suggests not only that regions which appear devoid of immunodominant epitopes may instead contain cryptic or subdominant epitopes, but also that the epitopes presented are influenced to a great extent by the nature of the MHC class II molecules. The HLA class II molecules are among the most polymorphic glycoproteins known in nature (Robinson et al., 2015). They play a crucial role in restricting the number of potential epitopes generated following the intracellular processing of antigens. MHC polymorphism is concentrated in the region encoding the peptide-binding groove, giving rise to a largely distinct set of epitopes that bind in a preferential manner to each distinct HLA allele. Murine studies have identified epitopes whose dominance or crypticity was dependent on the H-2 haplotype involved in presentation (Grewal et al., 1995; Moudgil et al., 1998; Sinha et al., 2007). It is therefore plausible that whilst none of the cryptic epitopes displayed a discernable pattern of binding across the HLA-DR alleles assayed, the different patterns of peptide binding affinity displayed by these LF epitopes, and specifically their ability to induce responses within the context of the whole LF protein, reflect the requirements of each MHC peptide binding cleft, which are nevertheless capable of inducing high avidity T cell responses.

The CD4+ T cell response across the distinct HLA alleles described in our results indicates that the immunodominant LF epitopes are overwhelmingly concentrated in domains II and IV, whilst domains I and III contain a small number of cryptic epitopes, presented in an HLA dependent manner. This contrasts with previous studies identifying B cell epitopes within the LF protein, which found that the levels of toxin neutralizing antibodies, proven to correlate with protection against lethal spore challenge (Baillie et al., 2008), are induced by domain I and II immunization. Domain III has also been found to be rich in binding sites for toxin neutralizing antibodies (Lim et al., 2005; Albrecht et al., 2007; Nguyen et al., 2009b). Indeed, the work by Nguyen et al. (2009a) indicated that domain III contained a number of structural B cell epitopes, making it extremely antigenic within this context. These results may reflect the nature of the conformational epitopes which, unlike linear CD4+ T cell epitopes, can be lost when domains are expressed as individual recombinant proteins. As CD4+ T cells play a crucial role in directing the adaptive immune response, not only through promoting the expansion of cytotoxic CD8+ T cells and maintaining the memory T cell population, but also through promoting B cell differentiation into plasma cells to produce neutralizing antibodies and assist memory B cells for a swift recall response. The identification of cryptic CD4+ T cell epitopes which may also be involved in initiating a neutralizing antibody response against LF would allow both the humoral and cellular immune system to provide robust protection to anthrax infection following immunization.

## Author Contributions

Conceived and designed the experiments: SA, RI, KC, BM, RB, and DA. Contributed reagents: SM, TH, BM, LB, and EW. Performed the experiments: SA, RI, KC, and BM. Analyzed the data: SA, RI, KC, and BM. Drafted the manuscript: SA, RI, RB, and DA. All authors critically revised and approved the final version.

## Conflict of Interest Statement

The authors declare that the research was conducted in the absence of any commercial or financial relationships that could be construed as a potential conflict of interest.
